# High rate of potentially inappropriate medication use in older people: a case–control study

**DOI:** 10.1007/s11357-024-01274-1

**Published:** 2024-07-09

**Authors:** András Érszegi, Dezső Csupor, Gabriella Bodó, Zsófia Engi, Muh. Akbar Bahar, Mária Matuz, Ria Benkő, Zoltán Pető, Réka Viola

**Affiliations:** 1 Faculty of Pharmacy, Institute of Clinical Pharmacy, University of Szeged, Szeged, Hungary; 2 Albert Szent-Györgyi Health Center, Central Pharmacy, University of Szeged, Szeged, Hungary; 3 Borsod-Abaúj-Zemplén County Central Hospital and University Teaching Hospital, Miskolc, Hungary; 4 Department of Pharmacy, Faculty of Pharmacy, Universitas Hasanuddin, Makassar, Indonesia; 5 Albert Szent-Györgyi Health Center, Department of Emergency Medicine, University of Szeged, Szeged, Hungary; 6 Institute for Translational Medicine, University of Pécs, Pécs, Hungary

**Keywords:** Potentially inappropriate medications, Falls, Older adults, EU(7)-PIM list, Hungary

## Abstract

Annually, 172 million fall events cause temporary or permanent impairment in older adults, and this number is increasing. Contributing factors that increase the risk for falls include age, polypharmacy, and malnutrition. This study evaluated medications mainly included in the EU(7)-PIM (potentially inappropriate medication) list. From March 21, 2022, to July 6, 2022, 945 patients who experienced a fall and visited the Department of Emergency Medicine at the Albert Szent-Györgyi Health Centre of the University of Szeged in Hungary. Data from 886 patients were collected (study group). The control group included 1364 patient data collected from three general practice in Hungary. The use of ≥ 2 EU(7)-PIM drugs was found to be associated with increased risk for falls (adjusted odds ratio [AOR], 1.38; 95% confidence interval [CI] 1.01–1.88). Piracetam (AOR, 1.81; 95% CI, 1.28–2.57) and trimetazidine (AOR, 1.62; 95% CI, 1.17–2.24) were associated with increased risk for falls. Doxazosin was associated with a low risk for falls (AOR, 0.59; 95% CI, 0.41–0.86). Tiapride (AOR, 3.54; 95% CI, 1.75–7.17), gliclazide (AOR, 1.57; 95% CI, 1.02–2.43), and vinpocetine (AOR, 1.95; 95% CI, 1.29–2.95) are not included in the EU(7)-PIM list; however, they are associated with increased risk for falls. Long-acting benzodiazepines (AOR, 1.79; 95% CI, 1.20–2.68), antidepressants (AOR, 1.89; 95% 95% CI, 1.37–2.61), serotonin–norepinephrine reuptake inhibitor (AOR, 2.82; 95% CI, 1.41–5.67; *p* < 0.01), and selective serotonin reuptake inhibitor (AOR, 1.88; 95% CI, 1.24–2.85) were also associated with increased risk for falls. However, Z-drugs were associated with a low risk for falls (AOR, 0.57; 95% CI, 0.36–0.92). With the help of this tool, trimetazidine and piracetam are filtered as EU(7)-PIM drugs associated with increased risk for falls.

## Introduction

Among older adults, falls are the second leading cause of injury-related mortality worldwide, accounting for 684,000 deaths annually [1]. Eighty percent of the fall-related fatalities occur in low- and middle-income countries. Furthermore, injury-related mortality increased by 6% between 2000 and 2019; however, fall-related deaths increased by 53% [2]. Globally, 172 million falls result in temporary or permanent impairment, placing a significant burden on the healthcare system [3]. Falls are the cause of approximately 38 million disability-adjusted life years annually, and this number is rising steadily [4]. Age is one of the several contributing factors to the increasing incidence of falls, as fall-related fatalities are most prevalent among older adults [5]. Falls exert a huge burden on the healthcare system because injuries such as hip fractures require expensive treatment (hospitalization and possible complications) [6].

Many studies and meta-analyses have focused on falls and reported various risk factors. These risk factors are related to demographics, physical and mental health, medical and socioeconomic status, environment, and behavior (e.g., inappropriate footwear, lack of exercise, and excess alcohol intake), which influence the risk for falls among older people. Parkinson’s disease, arthritis, impaired visual cognition, reduced mobility, balance problems, and depression significantly increase the risk for falls among the older population [7, 8]. In a systematic review and meta-analysis, which included 34 articles, older age, polypharmacy (use of ≥ 5 drugs), malnutrition, single status, living in a rural area, smoking, and alcohol consumption significantly increased the risk for falls among older adults. Furthermore, people with cardiac disease, hypertension, frailty, previous history of falls, and pain had a higher risk for falls than individuals without such comorbidities [9]. In a review of 18 studies involving 65,624 older people, Lu Shao et al. showed that female sex, dementia, insomnia, vertigo, walking aids, gait, poor balance, impaired activity of daily living, hearing problems, and use of antidepressants, anxiolytics, benzodiazepines, and antipsychotics were potential risk factors for falls in nursing home residents [10].

Many risk factors increase the risk of falls among older people, and medications are among these factors. For this reason, medication reconciliation is crucial for older people to prevent the intake of drugs that increase the risk of falls. In many studies, tools and criteria have been employed to inhibit the use of potentially inappropriate medications (PIM) for older adults (e.g., Beers list, [11] PRISCUS list, [12] START-STOPP criteria, [13] FORTA list, [14] and EU(7)-PIM list [15]). To identify and evaluate drugs that can increase the risk for falls, the EU(7)-PIM list, which summarizes medications that are not recommended for older people, was used in this study.

A systematic review and meta-analysis investigated drug groups and established that nonsteroidal anti-inflammatory drugs (NSAIDs), opioids, loop diuretics, BDZs, ADs, and antipsychotics may increase the risk for falls, which are categorized as fall risk-increasing drugs [16–18].

This study aimed to assess whether the EU(7)-PIM list can filter drugs that increase the risk for falls, analyze more PIM drug groups and individual active substances, determine whether they affect the risk for falls, and show the circumstances, time, and consequences of falls.

## Methods

### Study design

**Fig. 1:**
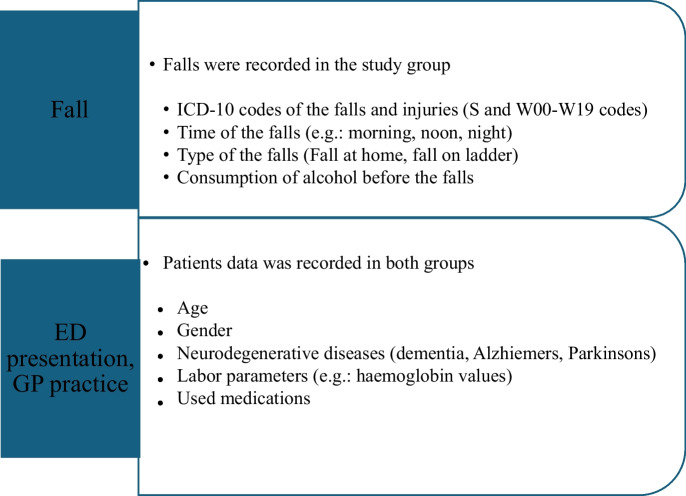
Flow chart of the collected data in the study and control group

This case–control study was performed in accordance with the STROBE statement for case–control studies [19]. It is based on patient data, which were collected from the eMedSol Hospital Information System (HIS) between March 21, 2022, and July 6, 2022, among older adults (aged > 65 years) who fell and visited the Department of Emergency Medicine at the Albert Szent-Györgyi Health Centre of the University of Szeged (ED). Falls were grouped by the time of occurrence, type of the falls, injuries after a fall by ICD-10 codes, and available laboratory parameters (anemia based on hemoglobin values: < 120 g/L in women and < 130 g/L in men) were collected. Further collected data are presented in Fig. 1. Data from three general practices (GP) were used as the control group: two from Szeged, Hungary, and one from Szatymaz, Hungary.

### Patients

The inclusion criteria for the study group were diagnosis with ICD-10 codes S or W00-W19 at the ED in Szeged and age at least 65 years. The eligibility requirements of the control group included being at least 65 years old; older people who had fallen during the previous year were excluded from the control group.

### Medications

The medication history was collected from the HIS, and if the patient’s medication history was not documented, patients were phoned to collect information about the prescribed drugs. The patients who could not be reached after three attempts were excluded. Only the prescribed medications were collected because OTC is not recorded in medical systems. The prescribed medications were categorized using the WHO Collaborating Centre for Drug Statistics Methodology (ATC code system). Polypharmacy was defined as the use of ≥ 5 active substances. Some active substances that are used in Hungary are not commonly used in other European countries. We considered some of them as PIMs because other active substances in the same drug group are included in the EU(7)-PIM list (e.g., BDs (cinolazepam), antipsychotics for long-term use, and tiapride). Other drugs (e.g., vinpocetine) are not unequivocally a PIM; however, based on their whole profile (side effects, efficacy, and safety), they were considered as a PIM. Only drugs used > 2.50% in the study group were analyzed individually. This cutoff value was chosen to avoid small-number bias. Moreover, drug groups (e.g., BDs and ADs) were also analyzed. The characteristics of the medications selected for statistical analysis are listed in Tables 1 and 2.

**Table 1:** Aspects for including active substances in the statistical analysis

Active substance included for further statistical analysis
Its pharmaceutical form is either per os or transdermal or parenteral
It is EU(7)-PIM listed, either part of other PIM lists or can be considered as a PIM drug
It is EU(7)-PIM listed and used by at least 2.50% of the patients in the study group
It is EU(7)-PIM listed and used in the study and control group too

**Table 2:** Aspects for excluding active substances from the statistical analysis

Active substance excluded from further statistical analysis
If its pharmaceutical form is either eyedrops or nose sprays, or it is for inhalation, or other externally used (e.g., creams, ointments)
It is not EU(7)-PIM listed, not part of other PIM lists, and cannot be considered as a PIM drug
It is EU(7)-PIM listed, but it is used by less than 2.50% of the patients in the study group and cannot be combined with other PIMs to make a group
It is dose dependent to be on the EU(7)-PIM list (e.g., spironolactone > 25 mg/day) (We have no information about dosages)
It is EU(7)-PIM listed, but not used in the study or control group

### Statistical analysis

Descriptive statistics were employed to summarize the baseline characteristics of the patients, presenting categorical data as numbers and percentages. The chi‐square test was utilized to compare the distribution of covariates between the control (patients without a history of falls) and study (patients with a history of falls) groups. Covariates included age (years); sex; presence of Alzheimer’s disease, Parkinson’s disease, or dementia; and polypharmacy (≥ 5 drugs). Covariates showing significant differences (*p* < 0.05) were included in the multivariate logistic regression model to determine the adjusted odds ratio (AOR) as a measure of association between PIM use and incidence of falls. In addition, the 95% confidence interval (CI) was calculated for each adjusted odds ratio [OR]. A statistically significant association was indicated by a *p*-value of < 0.05 and a 95% CI that did not include one. IBM SPSS Statistics for Windows version 29.0.1.0 (IBM Corp., Armonk, NY, USA) was used for the statistical analysis.

## Results

### Demographics

**Fig. 2:**
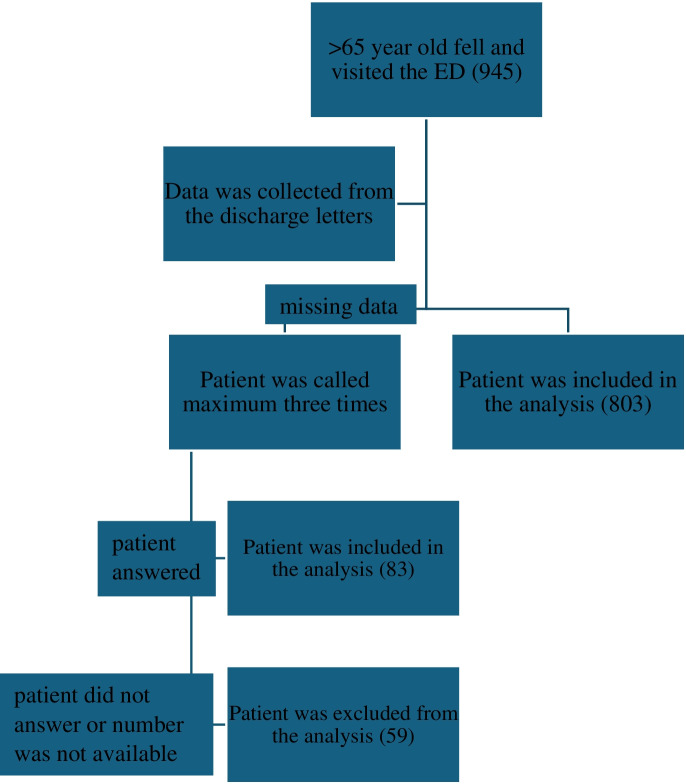
Flowchart of the collected data

Between March 21, 2022, and July 6, 2022, 945 older people were brought to the ED because of falls. Of these patients, we gathered data from 886 (93.76%) (study group) and excluded the remaining 59. Moreover, data from 83 older patients were collected by phone (9.37%) (Fig. 2). From the three GP practices, data from 1364 older patients were collected (control group). The baseline characteristics of the two groups are shown in Table 3.

**Table 3:** Comparison of baseline characteristics of the control (*n* = 1364) and study group (*n* = 886)

Variables	Total patients *n* (%)	Control group *n* (%)	Study group *n* (%)	*p*-value
Incidence of fall				
No fall	1364 (60.6)			
Fall	886 (39.4)			
Age in years				
65–75	1154 (51.3)	847 (62.1)	307 (34.7)	
> 75	1096 (48.7)	517 (37.9)	579 (65.3)	< 0.01
Sex				
Male	811 (36)	543 (39.8)	268 (30.2)	
Female	1439 (64)	821 (60.2)	618 (69.8)	< 0.01
Alzheimer				
No	2199 (97.7)	1355 (99.3)	844 (95.3)	
Yes	51 (2.3)	9 (0.7)	42 (4.7)	< 0.01
Parkinson				
No	2178 (96.8)	1322 (96.9)	856 (96.6)	
Yes	72 (3.2)	42 (3.1)	30 (3.4)	0.69
Dementia				
No	2115 (94)	1330 (97.5)	785 (88.6)	
Yes	135 (6)	34 (2.5)	101 (11.4)	< 0.01
Polypharmacy (≥ 5 drugs)				
No	838 (37.2)	550 (40.3)	288 (32.5)	
Yes	1412 (62.8)	814 (59.7)	598 (67.5)	< 0.01

Variables with *p* < 0.05 will enter multivariate models

**Fig. 3:**
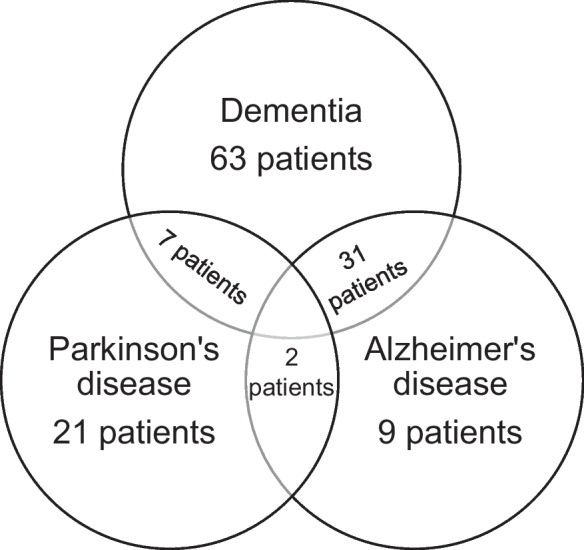
Venn diagram of the neurodegenerative diseases in the study group

Specifically, 113 (12.75%) patients lived in nursing homes and 195 (22.01%) needed admission to the traumatology department because of injuries. Moreover, 37 (4.18%) patients consumed alcohol before the fall event. Laboratory parameters were measured in 332 patients who presented at the ED after a fall. Based on hemoglobin values, 130 patients in the study group had anemia. In the control group, 36 of the 194 patients had anemia based on recent (but not earlier than 1 year) laboratory testing. Because of missing data in the control group, the result was not comprehensible in the case of anemia. The proportion of women aged > 75 years was significantly higher in the study group than in the control group (*p* < 0.01). The proportion of patients with dementia and Alzheimer’s disease was significantly higher in the study group than in the control group (*p* < 0.01). However, the distribution of patients with Parkinson’s disease was comparable between the study and control groups. Dementia was the most prevalent neurodegenerative condition in the study group, followed by Parkinson’s and Alzheimer’s diseases. Dementia was associated with Alzheimer’s disease in 30.69% of the patients, whereas 6.93% of the patients with dementia also suffered from Parkinson’s disease. Further data are presented in Fig. 3. The proportion of patients with polypharmacy was significantly higher in the study group than in the control group (*p* < 0.01).

### Falls

**Fig. 4:**
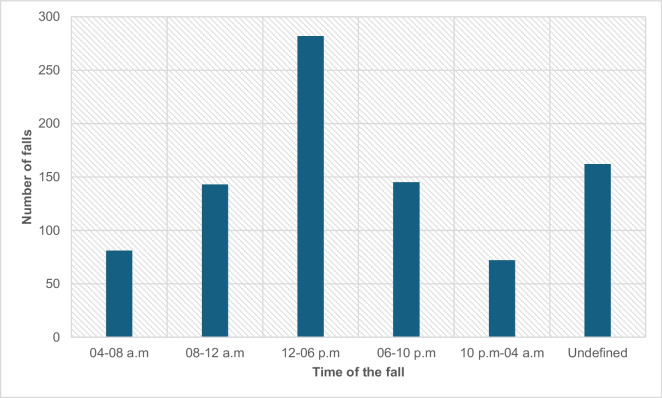
The time of the falls

Of the 886 fall events, 408 (46.05%) resulted in a bone fracture (ICD-10; S0200–S9290). Most falls (*n* = 282) occurred at noon (12 p.m.–06 p.m.). However, in many cases, the time that the fall occurred was not recorded (162 cases). Figure 4 shows the time of the occurrence of falls.

**Fig. 5:**
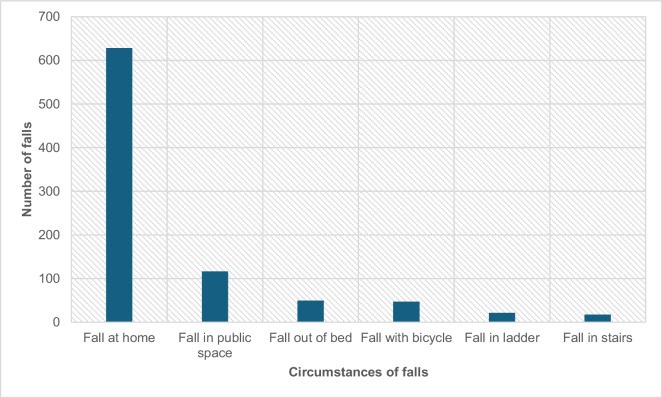
Circumstances of the falls, based on final reports

The three most common types of falls, according to the reported ICD-10 codes, were unspecified falls (W19; 62.64%); falls on the same level from slipping, tripping, and stumbling (W01; 15.24%); and falling out of bed (W06; 3.05%). According to the discharge letters, 629 fall events (slipping, tripping, stumbling, or not specified) occurred at the patient’s home during activity (70.99%), 116 (13.09) occurred in a public place, and 49 (5.53%) occurred when the patient fell from bed (Fig. 5).

Crushing skull injuries (S071; 38.60%), crushing injury of the hip (S770; 15.80%), and open scalp wounds (S010; 10.72%) were the three most common injuries after a fall. The most common injuries are presented in Table 4. Regarding the types of injuries, the three most common were head crushing injury (S07; 43.91%), crushing injury of the hip and thigh (S77; 16.82%), and an open wound on the head (S01; 15.12%).

**Table 4:** Most common injuries after fall

ICD-10 codes	Meaning of the code	Number of patients
**S07**	**Crushing injury of the head**	**389**
S071	Crushing injury of skull	342
S070	Crushing injury of face	30
**S77**	**Crushing injury of hip and thigh**	**149**
S770	Crushing injury of hip	140
**S01**	**Open wound in the head**	**134**
S010	Open wound of scalp	95
S014	Open wound of cheek and temporomandibular area	28
**S80**	**Superficial injury of the lower leg**	**132**
S800	Contusion of knee	104
S801	Contusion of other and unspecified parts of lower leg	13
**S20**	**Superficial injury of thorax**	**106**
S202	Contusion of thorax	74
S204	Other superficial injuries of back wall of thorax	29
**S72**	**Fracture of the femur**	**107**
S721	Pertrochanteric fracture	46
S720	Fracture of neck of femur	42
S723	Fracture of shaft of femur	9
S722	Subtrochanteric fracture	8

### Medications associated with an increased risk of falling

We gathered the medication data of 886 older people in the study group. These medications were compared with those of the control group (*n* = 1364). The frequency of EU(7)-PIM drug use is presented in Table 5. Patients taking two EU(7)-PIM drugs (adjusted odds ratio [AOR], 1.38; 95% confidence interval [CI], 1.01–1.88; *p* = 0 0.04) and three EU(7)-PIM drugs (AOR, 1.64; 95% CI, 1.20–2.24; *p* < 0.01) had significantly higher risk of falling than those not taking EU(7)-PIM drugs. In the group aged 65–75 years, if only one EU(7)-PIM drug was used, the risk of fall was not significantly different from those not using a EU(7)-PIM drug. However, the use of two (AOR, 2.09; 95% CI, 1.32–3.29; *p* < 0.01) and ≥ 3 EU(7)-PIM drugs (AOR, 2.20; 95% CI, 1.36–3.56; *p* < 0.01) were significantly associated with the incidence of fall. In addition, in the group aged ≥ 75 years, the use of EU(7)-PIM drugs was not significantly associated with the incidence of falls. Older men and women using ≥ 3 EU(7)-PIM drugs had significantly higher (male: AOR, 1.72; 95% CI, 1.03–2.87; *p* = 0.04; female: AOR, 1.60; 95% CI, 1.08–2.38; *p* = 0.02) risk of fall than those not using EU(7)-PIM drugs.

**Table 5:** Association between the used number of EU(7)-PIM drugs and the incidence of falls

Variable	Total patients, *n* (%)	Control group, *n* (%)	Study group, *n* (%)	Unadjusted OR (95% CI)	*p*-value	Adjusted OR* (95% CI)	*p*-value
Overall analysis							
PIM drugs							
No EU(7)-PIM drug	700 (31.1)	474 (34.8)	226 (25.5)	Reference		Reference	
1 EU(7)-PIM drug	547 (24.3)	343 (25.1)	204 (23)	1.25 (0.99–1.58)	0.07	1.24 (0.95–1.62)	0.12
2 EU(7)-PIM drugs	436 (19.4)	256 (18.8)	180 (20.3)	1.48 (1.15–1.89)	< 0.01	1.38 (1.01–1.88)	0.04
≥ 3 EU(7)-PIM drugs	567 (25.2)	291 (21.3)	276 (31.2)	1.99 (1.58–2.50)	< 0.01	1.64 (1.20–2.24)	< 0.01
Age groups							
*Age 65–75 years*							
PIM drugs							
No EU(7)-PIM drug	439 (19.5)	337 (39.8)	102 (33.2)	Reference			
1 EU(7)-PIM drug	310 (13.8)	229 (27)	81 (26.4)	1.17 (0.84–1.64)	0.36	1.42 (0.97–2.07)	0.07
2 EU(7)-PIM drugs	198 (8.8)	138 (16.3)	60 (19.5)	1.44 (0.99–2.09)	0.06	2.09 (1.32–3.29)	< 0.01
≥ 3 EU(7)-PIM drugs	207 (9.2)	143 (16.9)	64 (20.8)	1.48 (1.02–2.14)	0.04	2.20 (1.36–3.56)	< 0.01
*Age* > *75 years*							
PIM drugs							
No EU(7)-PIM drug	261 (11.6)	137 (26.5)	124 (21.4)	Reference		Reference	
1 EU(7)-PIM drug	237 (10.5)	114 (22.1)	123 (21.2)	1.19 (0.84–1.69)	0.33	1.08 (0.73–1.59)	0.70
2 EU(7)-PIM drugs	238 (10.6)	118 (22.8)	120 (20.7)	1.12 (0.79–1.60)	0.52	0.94 (0.61–1.44)	0.78
≥ 3 EU(7)-PIM drugs	360 (16.0)	148 (28.6)	212 (36.6)	1.58 (1.15–2.18)	< 0.01	1.21 (0.79–1.84)	0.39
Sex							
* Male*							
PIM drugs							
No EU(7)-PIM drug	278 (12.35)	195 (35.9)	83 (31)	Reference			
1 EU(7)-PIM drug	200 (8.9)	143 (26.3)	57 (21.3)	0.94 (0.63–1.40)	0.75	0.97 (0.62–1.52)	0.90
2 EU(7)-PIM drugs	158 (7.0)	105 (19.3)	53 (19.8)	1.19 (0.78–1.80)	0.42	1.22 (0.73–2.03)	0.45
≥ 3 EU(7)-PIM drugs	175 (7.8)	100 (18.4)	75 (28)	1.76 (1.19–2.61)	< 0.01	1.72 (1.03–2.87)	0.04
* Female*							
PIM drugs							
No EU(7)-PIM drug	422 (18.8)	279 (34)	143 (23.1)	Reference		Reference	
1 EU(7)-PIM drug	347 (15.4)	200 (24.4)	147 (23.8)	1.43 (1.07–1.92)	0.02	1.40 (0.99–1.97)	0.05
2 EU(7)-PIM drugs	278 (12.35)	151 (18.4)	127 (20.6)	1.64 (1.20–2.24)	< 0.01	1.45 (0.98–2.16)	0.06
≥ 3 EU(7)-PIM drugs	392 (17.4)	191 (23.3)	201 (32.5)	2.05 (1.55–2.72)	< 0.01	1.60 (1.08–2.38)	0.02

*Adjusted for age, sex, Alzheimer’s disease, dementia, and polypharmacy

Drugs that were used > 2.50% in the study group are presented in Table 6. PIMs were generally used more frequently in the study group (except for doxazosin, human insulin, esomeprazole, diclofenac, and zolpidem). The incidence of fall was only associated significantly with the use of trimetazidine (AOR, 1.62; 95% CI, 1.17–2.24; *p* < 0.01), piracetam (AOR, 1.81; 95% CI, 1.28–2.57; *p* < 0.01), vinpocetine (AOR, 1.95; 95% CI, 1.29–2.95); *p* < 0.01), gliclazide (AOR, 1.57; 95% CI, 1.02–2.43; *p* = 0.04), and tiapride (AOR, 3.54; 95% CI, 1.75–7.17; *p* < 0.01). However, patients taking doxazosin had a lower risk for falls (AOR, 0.59; 95% CI, 0.41–0.86; *p* < 0.01) than those not taking it (Table 7).

**Table 6:** Active substances, which are included in the analysis (used more than 2.50% in the study group)

Active substance used	Prevalence in the study group (%) (*n* = 886)	Prevalence in the control group (%) (*n* = 1364)	EU(7)-PIM
Pantoprazole	32.73 (290)	26.24 (358)	YES
Amlodipine	32.62 (289)	30.71 (419)	NO
Perindopril	30.70 (272)	30.63 (418)	NO
Acetylsalicylic acid	23.36 (207)	21.71 (296)	NO
Potassium chloride	20.32 (180)	10.86 (148)	NO
Alprazolam	19.07 (169)	16.30 (222)	YES
Cholecalciferol	18.96 (168)	21.49 (293)	NO
Indapamide	18.17 (161)	25.43 (347)	NO
Furosemide	17.83 (158)	12.49 (170)	NO
Metoprolol	17.38 (154)	15.17 (207)	NO
Bisoprolol	15.58 (138)	16.36 (223)	NO
Metformin	15.24 (135)	19.93 (272)	NO
Atorvastatin	14.00 (124)	15.54 (212)	NO
Ramipril	12.75 (113)	12.19 (166)	NO
Allopurinol	12.64 (112)	17.25 (235)	NO
Rosuvastatin	12.19 (108)	17.10 (233)	NO
Magnesium	12.30 (109)	8.55 (117)	NO
Clopidogrel	11.51 (102)	9.22 (126)	NO
Trimetazidine	11.06 (98)	6.33 (86)	YES
Piracetam	10.94 (97)	4.62 (63)	YES
Hydrochlorothiazide	10.61 (94)	22.08 (301)	NO
Rilmenidine	10.50 (93)	8.91 (122)	YES
Levotiroxin	10.50 (93)	8.77 (120)	NO
Nebivolol	9.93 (88)	10.86 (148)	NO
Famotidin	8.24 (78)	7.02 (96)	YES
Vinpocetin	7.79 (69)	3.12 (43)	Considered it PIM
Calcium	7.34 (65)	9.67 (132)	NO
Apixaban	7.00 (62)	5.65 (77)	YES
Nitroglicerin	6.32 (56)	2.83 (39)	NO
Carvedilol	6.09 (54)	4.53 (62)	NO
Doxazosin	5.98 (53)	8.12 (111)	YES
Valsartan	5.87 (52)	8.03 (110)	NO
Spironolacton	5.76 (51)	2.90 (40)	NO
Betahistin	5.76 (51)	3.42 (47)	PRISCUS listed
Lercanidipine	5.53 (49)	4.76 (65)	NO
Gliclazide	5.42 (48)	3.57 (49)	PRISCUS listed
Tramadol	5.19 (46)	3.34 (46)	YES
Tiapride	5.08 (45)	0.82 (11)	PRISCUS listed
Telmisartan	4.97 (44)	8.48 (115)	NO
Tamsulosin	4.06 (36)	5.72 (78)	NO
Pentoxifylline	3.95 (35)	2.63 (36)	YES
Enalapril	3.72 (33)	4.76 (65)	NO
Human insulin	3.61 (32)	4.76 (65)	YES
Clopamid	3.50 (31)	0.74 (10)	NO
Isosorbid-mononitrate	3.50 (31)	2.16 (29)	NO
Thioctic acid	3.27 (29)	3.94 (54)	NO
Esomeprazol	3.04 (27)	4.31 (59)	YES
Enoxaparin	2.82 (25)	1.26 (17)	NO
Clonazepam	2.82 (25)	1.64 (22)	YES
Acenocoumarol	2.71 (24)	1.64 (22)	YES
Lisinopril	2.71 (24)	2.20 (30)	NO
Nicergoline	2.60 (23)	1.17 (16)	YES
Ascorbic acid	2.60 (23)	0.51 (7)	NO

**Table 7:** Association between PIM drugs and the incidence of falls

List of PIM drugs	Total patients, *n* (%)	Control group, *n* (%)	Study group, *n* (%)	Unadjusted OR (95% CI)	*p*-value	Adjusted OR*** (95% CI)	*p*-value
*Pantoprazole*							
No	1602 (71.2)	1006 (73.8)	596 (67.3)	Reference		Reference	
Yes	648 (28.8)	358 (26.2)	290 (32.7)	1.37 (1.14–1.65)	< 0.01	1.20 (0.97–1.48)	0.09
*Alprazolam*							
No	1858 (82.6)	1141 (83.7)	717 (80.9)	Reference		Reference	
Yes	392 (17.4)	223 (16.3)	169 (19.1)	1.21 (0.97–1.50)	0.10	1.01 (0.79–1.28)	0.95
*Rilmenidine*							
No	2036 (90.5)	1243 (91.1)	793 (89.5)	Reference		Reference	
Yes	214 (9.5)	121 (8.9)	93 (10.5)	1.21 (0.91–1.60)	0.20	1.04 (0.76–1.42)	0.80
*Trimetazidine*							
No	2066 (91.8)	1278 (93.7)	788 (88.9)	Reference		Reference	
Yes	184 (8.2)	86 (6.3)	98 (11.1)	1.85 (1.37–2.50)	< 0.01	1.62 (1.17–2.24)	< 0.01
*Famotidine*							
No	2082 (92.5)	1269 (93)	813 (91.8)	Reference		Reference	
Yes	168 (7.5)	95 (7)	73 (8.2)	1.20 (0.87–1.65)	0.26	1.20 (0.86–1.68)	0.29
*Doxazosin*							
No	2086 (92.7)	1253 (91.9)	833 (94)	Reference		Reference	
Yes	164 (7.3)	111 (8.1)	53 (6)	0.72 (0.51–1.01)	0.06	0.59 (0.41–0.86)	< 0.01
*Piracetam*							
No	2090 (92.9)	1301 (95.4)	789 (89.1)	Reference		Reference	
Yes	160 (7.1)	63 (4.6)	97 (10.9)	2.54 (1.83–3.53)	< 0.01	1.81 (1.28–2.57)	< 0.01
*Apixaban*							
No	2112 (93.9)	1288 (94.4)	824 (93)	Reference		Reference	
Yes	138 (6.1)	76 (5.6)	62 (7)	1.28 (0.90–1.80)	0.17	0.96 (0.66–1.39)	0.83
Vinpocetine*							
No	2139 (95.1)	1322 (96.9)	817 (92.2)	Reference		Reference	
Yes	111 (4.9)	42 (3.1)	69 (7.8)	2.66 (1.79–3.94)	< 0.01	1.95 (1.29–2.95)	< 0.01
Betahistine**							
No	2153 (95.7)	1318 (96.6)	835 (94.2)	Reference		Reference	
Yes	97 (4.3)	46 (3.4)	51 (5.8)	1.75 (1.16–2.63)	< 0.01	1.17 (0.76–1.82)	0.47
Gliclazide**							
No	2154 (95.7)	1316 (96.5)	838 (94.6)	Reference		Reference	
Yes	96 (4.3)	48 (3.5)	48 (5.4)	1.57 (1.04–2.37)	0.03	1.57 (1.02–2.43)	0.04
*Insulin human*							
No	2154 (95.7)	1300 (95.3)	854 (96.4)	Reference		Reference	
Yes	96 (4.3)	64 (4.7)	32 (3.6)	0.76 (0.49–1.17)	0.22	0.73 (0.46–1.16)	0.19
*Tramadol*							
No	2159 (96.0)	1319 (96.7)	840 (94.8)	Reference		Reference	
Yes	91 (4.0)	45 (3.3)	46 (5.2)	1.61 (1.06–2.44)	0.03	1.34 (0.86–2.10)	0.19
*Esomeprazole*							
No	2164 (96.2)	1305 (95.7)	859 (97)	Reference		Reference	
Yes	86 (3.8)	59 (4.3)	27 (3)	0.69 (0.44–1.11)	0.12	0.72 (0.44–1.18)	0.19
*Pentoxifylline*							
No	2179 (96.8)	1328 (97.4)	851 (96)	Reference		Reference	
Yes	71 (3.2)	36 (2.6)	35 (4)	1.52 (0.95–2.44)	0.08	1.08 (0.65–1.79)	0.76
Tiapride**							
No	2194 (97.5)	1353 (99.2)	841 (94.9)	Reference		Reference	
Yes	56 (2.5)	11 (0.8)	45 (5.1)	6.58 (3.38–12.79)	< 0.01	3.54 (1.75–7.17)	< 0.01
*Zolpidem*							
No	2194 (97.5)	1327 (97.3)	867 (97.9)	Reference		Reference	
Yes	56 (2.5)	37 (2.7)	19 (2.1)	0.79 (0.45–1.38)	0.40	0.62 (0.34–1.13)	0.12
*Diclofenac*							
No	2196 (97.6)	1325 (97.1)	871 (98.3)	Reference		Reference	
Yes	54 (2.4)	39 (2.9)	15 (1.7)	0.59 (0.32–1.07)	0.08	0.57 (0.30–1.10)	0.09
*Clonazepam*							
No	2203 (97.9)	1342 (98.4)	861 (97.2)	Reference		Reference	
Yes	47 (2.1)	22 (1.6)	25 (2.8)	1.77 (0.99–3.16)	0.05	1.52 (0.82–2.80)	0.18
*Acenocoumarol*							
No	2204 (98.0)	1342 (98.4)	862 (97.3)	Reference		Reference	
Yes	46 (2.0)	22 (1.6)	24 (2.7)	1.70 (0.95–3.05)	0.08	1.33 (0.72–2.46)	0.37
*Nicergoline*							
No	2211 (98.3)	1348 (98.8)	863 (97.4)	Reference		Reference	
Yes	39 (1.7)	16 (1.2)	23 (2.6)	2.25 (1.18–4.27)	0.01	1.01 (0.49–2.09)	0.99

*Not part of the EU(7)-PIM list, but a potential PIM drug

**Not part of the EU(7)-PIM list, but part of the PRISCUS list

***Adjusted for age, sex, Alzheimer’s disease, dementia, and polypharmacy

Active substances were grouped, and their frequency of use was compared in the two groups (Table 8). The members of the groups are listed in Table 9. Patients taking LBDZs, ADs, SNRIs, and SSRIs had significantly higher (LBDZ: AOR, 1.79; 95% CI, 1.20–2.68; *p* < 0.01; AD: AOR, 1.89; 95% CI, 1.37–2.61; *p* < 0.01; SSRI: AOR, 1.88; 95% CI, 1.24–2.85; *p* < 0.01; SNRI: AOR, 2.82; 95% CI, 1.41–5.67; *p* < 0.01) risk for falls than those not taking these drugs. However, Z-drugs had a protective effect by lowering (AOR, 0.57; 95% CI, 0.36–0.92; *p* = 0.02) the risk for falls.

**Table 8:** Association between PIM drug groups and the incidence of fall

Variable	Total patients, *n* (%)	Control group, *n* (%)	Study group, *n* (%)	Unadjusted OR (95% CI)	*p*-value	Adjusted OR* (95% CI)	*p*-value
Benzodiazepines							
No	1753 (77.0)	1086 (79.6)	647 (73)	Reference		Reference	
Yes	517 (23.0)	278 (20.4)	239 (27)	1.44 (1.18–1.76)	< 0.01	1.22 (0.98–1.51)	0.08
Short-acting benzodiazepines							
No	1838 (81.7)	1133 (83.1)	705 (79.6)	Reference		Reference	
Yes	412 (18.3)	231 (16.9)	181 (20.4)	1.26 (1.02–1.56)	0.04	1.07 (0.85–1.36)	0.57
Long-acting benzodiazepines							
No	2135 (94.9)	1314 (96.3)	821 (92.7)	Reference		Reference	
Yes	115 (5.1)	50 (3.7)	65 (7.3)	2.08 (1.42–3.04)	< 0.01	1.79 (1.20–2.68)	< 0.01
Z-drugs							
No	2157 (95.9)	1301 (95.4)	856 (96.6)	Reference		Reference	
Yes	93 (4.1)	63 (4.6)	30 (3.4)	0.72 (0.47–1.13)	0.15	0.57 (0.36–0.92)	0.02
Antidepressants							
No	2054 (91.3)	1281 (93.9)	773 (87.2)	Reference		Reference	
Yes	196 (8.7)	83 (6.1)	113 (12.8)	2.26 (1.68–3.04)	< 0.01	1.89 (1.37–2.61)	< 0.01
SSRI							
No	2141 (95.2)	1318 (96.6)	823 (92.9)	Reference		Reference	
Yes	109 (4.8)	46 (3.4)	63 (7.1)	2.19 (1.48–3.24)	< 0.01	1.88 (1.24–2.85)	< 0.01
SNRI							
No	2210 (98.2)	1350 (99)	860 (97.1)	Reference		Reference	
Yes	40 (1.8)	14 (1)	26 (2.9)	2.92 (1.51–5.61)	< 0.01	2.82 (1.41–5.67)	< 0.01
Beta-blockers							
No	2120 (94.2)	1294 (94.9)	826 (93.2)	Reference		Reference	
Yes	130 (5.8)	70 (5.1)	60 (6.8)	1.34 (0.94–1.92)	0.10	1.37 (0.94–1.99)	0.09
PPI							
No	1447 (64.3)	905 (66.3)	542 (61.2)	Reference			
Yes	803 (35.7)	459 (33.7)	344 (38.8)	1.25 (1.05–1.49)	0.01	0.93 (0.76–1.14	0.51

*Adjusted for age, sex, Alzheimer’s disease, dementia, and polypharmacy

**Table 9:** Members of the analyzed drug groups

Benzodiazepines	Antidepressants	Proton pump inhibitors	Z-drugs	Beta-blockers
Alprazolam**	Agomelatine	Esomeprazole	Zolpidem	Carvedilol*****
Brotizolam**	Amitriptyline	Lansoprazole	Zopiclone	Propranolol
Clonazepam*	Bupropion	Omeprazole	Eszopiclone	Sotalol
Cinolazepam**	Citalopram***	Pantoprazole		
Chlordiazepoxide*	Duloxetine****	Rabeprazole		
Clobazam*	Escitalopram***			
Diazepam*	Mianserin			
Medazepam*	Mirtazapine			
Midazolam**	Moclobemide			
Nitrazepam*	Paroxetine***			
Temazepam**	Sertraline***			
Tofisopam**	Trazodone			
	Tianeptine			
	Venlafaxine****			
	Vortioxetine			

*Long-acting BDZ

**Short-acting BDZ

***SSRI

****SNRI

*****Questionable EU(7)-PIM

## Discussion

This study not only revealed associations between drug groups and risk for falls but also provided data for individual active substances. It also provides information regarding the circumstances of falls and the types of resulting injuries. Most falls occurred at noon, and the vast majority of the older fell at home. This indicates that most fall events could be prevented or the severity of the injuries could be reduced by making their homes safer [20]. Previous research has shown that falls place a significant burden on older people who sustained fall-related events and on the healthcare system because of the high cost of treating related injuries [6].

This study revealed that the most prevalent injuries occur on the head and hip–thigh region. According to a 2020 study, the most common types of fractures include hip and spine fractures and head traumas [21]. Moreover, being a woman, age > 75 years, presence of dementia and Alzheimer’s disease, and polypharmacy can be risk factors for falls, which correlate with the results of previous studies [7–10]. The difference in the use of > 2 EU(7)-PIM drugs was significantly higher in the study group, which indicates that the use of at least two EU(7)-PIM drugs may increase the risk for falls. However, the use of > 3 EU(7)-PIM drugs was not associated with an increased risk of falls in the group aged > 75 years but in the group aged 65–75 years. Based on our results, pantoprazole, betahistine, tramadol, [18] clonazepam, [16] and nicergoline may be associated with increased risk for falls; however, the association was stronger in cases of piracetam and trimetazidine. Piracetam is used as a cognitive enhancer in Hungary, mostly in older people with cognitive impairment. However, it is a EU(7)-PIM drug because its effectiveness in cognitive impairment is not supported by clinical evidence. Common (> 1/100– ≤ 1/10) side effects (such as hyperkinesis and nervousness) of piracetam may increase the risk for falls, which was confirmed by our findings [22]. Parkinson’s-like symptoms are one of the side effects of trimetazidine, which may increase the risk for falls, and this is supported by our results [23]. Gliclazide and tiapride are not EU(7)-PIM drugs; however, they are associated with an increased risk for falls. Moreover, they both are included in the PRISCUS list, and other sulfanylureas (glibenclamide, glipizide, and glimepiride) and atypical antipsychotics are included in the EU(7)-PIM list. Vinpocetine is not included in either PIM list; however, we considered it a PIM because its effectiveness in its indication is not clinically proven according to a Cochrane review [24]; moreover, vinpocetine is a derivative of vincamine, which is included in the EU(7)-PIM list. One of the indications of vinpocetine is vascular dementia; however, Hungarian guidelines [25] question its effectiveness in this condition. It was used significantly more in the study group than in the control group; therefore, it is not only effective in its indicated use but may increase the risk of falls. Thus, we marked it as a PIM.

Although doxazosin is classified in the EU(7)-PIM list as a drug that increases the risk for falls because of its side effects (somnolence and vertigo), in the present study, those taking doxazosin had a lower risk for falls [26]. Furthermore, rilmenidine, another antihypertensive drug on the EU(7)-PIM list, did not increase the risk of falls in our investigation.

Previous studies, systematic reviews, and meta-analyses have shown that long-acting and short-acting BDZs, ADs, and antipsychotics are related to an increased risk for falls [17]. In the present study, PPIs and SBDZs may be associated with increased risk for falls; however, in the case of LBDZs, ADs, SSRIs, and SNRIs, this association appears stronger. With the use of Z-drugs, the risk for falls was lower; therefore, as hypnotics, they appear to be a better choice than long- and short-acting BDZs as regards the risk for falls.

## Conclusions

This study focused on the EU(7)-PIM list to find active substances that may increase the risk for falls; however, adjustments should always be made because the medication lists used differ by country. The use of > 2 EU(7)-PIM drugs appears to be an important factor in increasing the risk of falls. Piracetam, trimetazidine, which are EU(7)-PIM drugs, and gliclazide, tiapride and vinpocetine, LBDZs, ADs, SSRIs, and SNRIs are associated with increased risk for falls. However, Z-drugs appear to be much safer hypnotics than benzodiazepines. Doxazosin is associated with a lower risk for falls; however, this association should be investigated further.

In conclusion, the EU(7)-PIM list appears to be an appropriate tool for screening drugs that increase the risk of falls; however, the list should be adapted among countries based on their medicine lists.

## Limitations

Although we gathered information about the presence of neurodegenerative diseases, we do not have information about other morbidities (e.g., cardiovascular diseases). In addition, not over 1-year-old blood tests were available in only some participants of the control group, which indicates that only a few older patients had updated laboratory information. Therefore, data were not enough for statistical analysis. Physical variables (activity level) and other physical parameters (height and weight) were not recorded in this study. The effect of some of the active substances on the risk for falls could not be assessed because of the lack of data on the use of OTC medicines (e.g., NSAIDs) (Table 10).

**Table 10:** List of drugs that were analyzed in the study and available as OTC in Hungary

List of drugs that are available as OTC	EU (7)-PIM
Pantoprazole	YES
Acetylsalicylic acid	NO
Cholecalciferol	NO
Magnesium	NO
Piracetam	YES
Famotidin	YES
Calcium	NO
Esomeprazole	YES
Ascorbic acid	NO

## Data Availability

The datasets analyzed during the current study are available from the corresponding author on reasonable request.

## Abbreviations

AOR: Adjusted odds ratio
OR: Odds ratio
CI: Confidence interval
PPI: Proton pump inhibitor
SSRI: Selective serotonin receptor inhibitor
SNRI: Serotonin–norepinephrine receptor inhibitor
BDZ: Benzodiazepine
LBDZ: Long-acting benzodiazepine
SBDZ: Short-acting benzodiazepine
AD: Antidepressant
NSAID: Nonsteroidal anti-inflammatory drugs
PIM: Potentially inappropriate medication
ER: Emergency room
ICD: International Classification of Diseases
HIS: Hospital Information System
OTC: Over-the-counter
WHO: World Health Organization
ATC: Anatomical therapeutic chemical
SPSS: Statistical Package for the Social Sciences
GP: General practice
